# Prescribing Pattern of Anti-Parkinson Drugs in Japan: A Trend Analysis from 2005 to 2010

**DOI:** 10.1371/journal.pone.0099021

**Published:** 2014-06-06

**Authors:** Sachiko Nakaoka, Tatsuro Ishizaki, Hisashi Urushihara, Toshihiko Satoh, Shunya Ikeda, Mitsutoshi Yamamoto, Takeo Nakayama

**Affiliations:** 1 Department of Health Informatics, Kyoto University School of Public Health, Kyoto, Japan; 2 Human Care Research Team, Tokyo Metropolitan Institute of Gerontology, Tokyo, Japan; 3 Division of Drug Development and Regulatory Science, Faculty of Pharmacy, Keio University, Tokyo, Japan; 4 School of Social Informatics, Aoyama Gakuin University, Kanagawa, Japan; 5 Department of Pharmaceutical Sciences, School of Pharmacy, International University of Health and Welfare, Tochigi, Japan; 6 Takamatsu Neurology Clinic, Kagawa, Japan; Chiba University Center for Forensic Mental Health, Japan

## Abstract

**Objective:**

Therapeutic options for Parkinson's disease mainly consist of L-dopa and dopamine agonists. However, in Japan, the product labeling of the ergot dopamine agonists, cabergoline and pergolide, was revised in April 2007 due to the risk of developing cardiac valvulopathy. Here, we describe the prescribing trends of anti-Parkinson drugs from 2005 through 2010 in Japan, and examined whether these trends changed after the drug safety measures in 2007.

**Methods and Patients:**

We used medical claim data from January 2005 to December 2010 for Parkinson's disease patients older than 30 years who were prescribed anti-Parkinson drugs. We calculated the proportion of patients prescribed each drug for each year, and compared the proportions of first-line drugs prescribed before and after April 2007. We also examined the prescription variations of cabergoline/pergolide users one year before or after April 2007.

**Results:**

L-dopa was the most frequently prescribed drug for Parkinson's disease (2005, 58%; 2010, 51%). The proportion of patients prescribed ergot dopamine agonists markedly decreased and non-ergot dopamine agonists increased after 2007. Among first-line drugs, the proportion of non-ergot agents increased after April 2007. Among 54 cabergoline/pergolide users, 24 (44%) discontinued these drugs, nine of whom switched to non-ergot agents.

**Conclusion:**

L-dopa was the mainstay of Parkinson's disease treatment between 2005 and 2010 in Japan. There was a decrease in ergot agents and an increase in non-ergot agents prescribed after the regulatory actions in 2007.

## Introduction

Parkinson's disease (PD) is a common neurodegenerative disorder associated with aging. In Japan, PD occurs with a prevalence of 109–167 and annual incidence of 10.3 (per 100,000 people) [1], [2]. In recent years, with the release of new anti-Parkinson drugs, treatment options for PD have increased. Recent drug utilization studies on the prescribing pattern of anti-Parkinson drugs up to 2005 showed that L-dopa was most frequently used, while dopamine agonist use had been increasing since 2000 [3]–[6]. According to the clinical practice guidelines for PD of the Japanese Society of Neurology and the National Institute for Health and Clinical Excellence, dopamine agonists are used as a monotherapy in early-stage PD to delay starting patients on L-dopa [7], [8]. Dopamine agonists are classified as ergot alkaloid derivatives, such as cabergoline, pergolide, and bromocriptine, or non-ergot agents, such as ropinirole, pramipexole, and talipexole. Since 2004, several comparative studies have reported an association between the prevalence of cardiac valvulopathy and the use of cabergoline or pergolide [9]–[11]. The product labels of these two drugs were revised in Japan according to an alert by the Ministry of Health, Labour and Welfare in April 2007 as follows: to restrict the use of these drugs to second-line treatment after the use of non-ergot agents, to add a contraindication against patients with a history of valvulopathy, and to recommend periodic echocardiograms to detect valvulopathy before and during medication [12], [13].

In general, regulatory risk minimization activities such as product label revision to include adverse events are taken to modify clinical practices. However, a previous study showed that the regulatory actions in April 2007 had no impact on the proportion of PD patients prescribed cabergoline or pergolide [14]. Furthermore, the long-term influence of these actions on prescriptions is still unknown, as are the prescribing trends since 2005, i.e., after new drugs were released and the valvulopathy risk of ergot dopamine agonists revealed [3].

To address these issues, we investigated prescribing trends of anti-Parkinson drugs from 2005 through 2010 with special reference to changes after the valvulopathy drug safety measures taken in 2007.

## Methods

### Data sources

We utilized a medical claim database provided by the Japan Medical Data Center Co., Ltd. (JMDC, Tokyo, Japan) [15]. The JMDC database, which is among the limited number of databases available in Japan, has been used in several studies [16]–[22]. This database contains medical claim data from approximately 320,000 people in 2005 and 1,000,000 people in 2010 from 20 corporate health insurance societies, of which enrollees were employees and their family members. The database population includes 3.3% of subscribers of the society-managed health insurance (30 million people) [23], accounting for 0.8% of the whole population of Japan (130 million people). The database provides monthly data on patient demographics, test orders, treatments, drugs prescribed, and medical facilities for ambulatory and inpatient care. Drugs are coded according to the Anatomical Classification of Pharmaceutical Products (ATC) of the European Pharmaceutical Market Research Association [24]. Diagnoses for medical claims are coded according to the International Statistical Classification of Diseases and Related Health Problems, 10th Revision Version for 2003 (ICD-10). Elderly people comprise only a minor fraction of the population represented in the database (enrollees aged 65 years or older in 2010, 1.2%) because subscribers of the society-managed corporate health insurance are mainly middle-aged people.

### Study subjects

We analyzed PD patients who were prescribed anti-Parkinson drugs between January 2005 and December 2010. Diagnosis of PD was defined as presence of the ICD-10 code for “Parkinson's disease” (G-20). Prescription of anti-Parkinson drugs was defined as presence of the ATC code for “anti-Parkinson drugs” (N04A). Patients younger than 30 years were excluded because they likely suffered from antipsychotic-induced parkinsonism, given that the mean age of PD onset is 62.7 years and 90.2% of PD patients are 40 years or older in the nationwide registry [25]. Exclusion criteria were adjusted as appropriate for the analytical purpose.

### Ethics statement

Because the medical claim data investigated in this study were de-identified before provision, this study was exempt from obtaining informed consent from individual patients according to the local ethical guidelines for epidemiological research. The study protocol was approved by the Kyoto University Graduate School and Faculty of Medicine Ethics Committee (No. E-1195).

### Definition of variables

The variables included gender, age, PD duration, prescribed anti-Parkinson drugs, first-line drug, and drugs that induce parkinsonism. PD duration was defined as the month from official diagnosis until the last month of recorded treatment of PD. The anti-Parkinson drugs included in each category are as follows. L-dopa includes levodopa alone and a combination of levodopa with a dopa-decarboxlyase inhibitor (benserazide and carbidopa). Ergot dopamine agonists include cabergoline, pergolide, and bromocriptine. Non-ergot dopamine agonists include pramipexole, ropinirole, and talipexole. Anticholinergics include biperiden, trihexyphenidyl, profenamine, piroheptine, and mazaticol. “Others” include selegiline, entacapone, amantadine, droxidopa, and zonisamide. First-line drug was defined as the anti-Parkinson drug initially prescribed to the patient in the same month or after the incidence of PD from July 2005 to December 2010. To exclude potential prevalent cases, incidence of PD was defined as the first diagnosis of PD after at least six months of untreated PD following registration in this database [26], [27]. Since the database contains no data prior to January 2005, patients who received the first PD diagnosis from January to June 2005 were excluded from first-line analysis, as they might have taken PD medication before the study period. Drugs that induce parkinsonism were defined as presence of the ATC code for “antipsychotics” (N05A), as well as dopamine antagonists such as metoclopramide, sulpiride, domperidone, and reserpine.

### Statistical analysis

The Cochran-Armitage trend test [28] was first used to assess the time trend of the annual proportion of patients prescribed each of the anti-Parkinson drugs and each drug category. Patients prescribed anti-Parkinson drugs together with drugs that induce parkinsonism in the same claim even once in one year were excluded from this analysis. The proportions of first-line drugs prescribed before and after April 2007 were compared using the chi-square test or Fisher's exact test. We termed the period from July 2005 to March 2007 as “pre-revision,” and from April 2007 to December 2010 as “post-revision.” The number of PD patients prescribed each first-line drug and each drug category during each period was calculated as a proportion of the number of all PD patients prescribed first-line drugs in that period. Patients who were prescribed first-line drugs together with drugs that induce parkinsonism in the same claim were excluded from this analysis. Finally, for patients prescribed cabergoline or pergolide between April 2006 and March 2008, i.e., one year before or after the regulatory actions in April 2007, we confirmed whether these drugs were continued until March 2008, or discontinued beforehand. Patients who discontinued cabergoline or pergolide were divided into the following three groups: switched to other anti-Parkinson drugs, continued other anti-Parkinson drugs that had been co-prescribed, and discontinued all anti-Parkinson drugs at once. The number of non-ergot agent users who switched to cabergoline or pergolide in the same period was also examined.

Data were analyzed using SPSS 16.0 for Windows (SPSS Inc., Chicago Illinois) or R version 2.13.2. All reported *P*-values were two-tailed, and the level of significance was defined as *P*<0.05, with Bonferroni correction for multiple hypothesis testing.

## Results

Among 2,875 people diagnosed with PD (G20) in the claim data between 2005 and 2010, 2,223 were prescribed anti-Parkinson drugs. Excluding people who were younger than 30 years old—because real PD is very unlikely, there were 1,849 patients (mean age, 50.0 years; standard deviation, 14.6), of whom 804 (43.5%) were men. Among them, 714 patients were included in the trend analysis, 414 patients in the first-line analysis, and 54 patients in the cabergoline/pergolide user analysis. Because the number of patients registered in the database increased approximately three-fold in 2010 (1,000,000 people) relative to 2005 (320,000 people), most patients were enrolled in the middle of the survey period.

**Figure 1:**
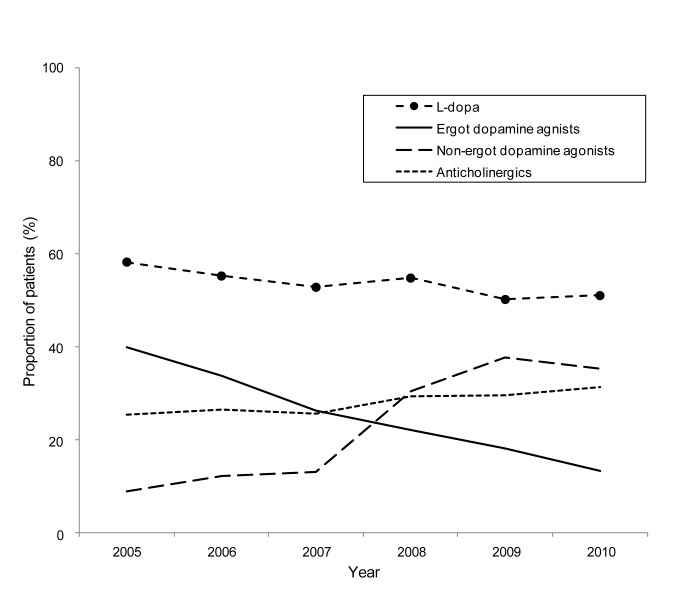
Annual plot for the proportion of Parkinson's disease patients prescribed each category of anti-Parkinson drugs. Ergot dopamine agonists decreased (*P*<0.001), and non-ergot dopamine agonists increased (*P*<0.001). With respect to L-dopa and anticholinergics, there was no significant change (*P* = 0.114, *P* = 0.106). Cochran-Armitage trend test was used to calculate P-values (statistical significance level was set at *P*<0.002 after Bonferroni correction).

Results of the trend analysis are as follows. Table 1 shows the characteristics of the target group each year and the annual proportion of patients prescribed each anti-Parkinson drug. The median age was 60–64 years from 2005 through 2008, but was 57 years in 2009 and 56 years in 2010. Proportions of men and women were almost equal for each year. The median disease duration was 24–38 months from 2005 to 2008, but 23 months in 2009 and 22 months in 2010. L-dopa was the most frequently prescribed drug throughout the study period (2005, 58.2%; 2010, 51.0%). The proportion of patients prescribed cabergoline decreased from 2005 to 2010 (*P*<0.001), whereas proportions of patients prescribed pramipexole, ropinirole, entacapone, and zonisamide, which were newly introduced, increased during this period (*P*<0.001). Figure 1 shows the time trend of the annual proportion of patients prescribed each category of anti-Parkinson drugs. L-dopa was the most frequently prescribed, followed by dopamine agonists. Significant changes in prescription patterns were observed, with a decrease in ergot dopamine agonists (2005, 40.0%; 2010,13.3%, *P*<0.001) and increase in non-ergot dopamine agonists (2005, 9.1%; 2010, 35.4%; *P*<0.001). Anticholinergics were prescribed to approximately 30% of patients each year.

**Table 1:** Proportions of Parkinson's Disease Patients Prescribed Anti-Parkinson Drugs.

	2005		2006		2007		2008		2009		2010		
	(n = 110) (%)		(n = 154) (%)		(n = 144) (%)		(n = 263) (%)		(n = 315) (%)		(n = 353) (%)		*P*-value c
Age, median, IQR	60, 53–70		63, 53–75		64, 52–76		60, 49–72		57, 47–63		56, 46–62		*-*
Gender (male), N (%)	55	(50.0)	71	(46.1)	68	(47.2)	133	(50.6)	163	(51.7)	180	(51.0)	
Parkinson's disease duration (months) median, IQR	24, 10–60		29, 11–62		38, 13–67		29, 10–67		23, 7–61		22, 7–52		*-*
L-dopa													
Levodopa^a^	64	(58.2)	85	(55.2)	76	(52.8)	144	(54.8)	158	(50.2)	180	(51.0)	0.114
Ergot dopamine agonists													
Cabergoline	25	(22.7)	34	(22.1)	25	(17.4)	30	(11.4)	29	(9.2)	22	(6.2)	<0.001
Pergolide	12	(10.9)	10	(6.5)	6	(4.2)	12	(4.6)	12	(3.8)	14	(4.0)	0.008
Bromocriptine	9	(8.2)	10	(6.5)	9	(6.3)	17	(6.5)	17	(5.4)	13	(3.7)	0.050
Non-ergot dopamine agonists													
Pramipexole	9	(8.2)	17	(11.0)	16	(11.1)	62	(23.6)	88	(27.9)	99	(28.0)	<0.001
Ropinirole	0	(0.0)	0	(0.0)	3	(2.1)	16	(6.1)	32	(10.2)	29	(8.2)	<0.001
Talipexole	1	(0.9)	2	(1.3)	2	(1.4)	9	(3.4)	5	(1.6)	4	(1.1)	0.989
Anticholinergics													
Trihexyphenidyl	21	(19.1)	32	(20.8)	31	(21.5)	53	(20.2)	67	(21.3)	88	(24.9)	0.174
Biperiden	7	(6.4)	9	(5.8)	7	(4.9)	21	(8.0)	25	(7.9)	22	(6.2)	0.647
Other anticholinergics^b^	0	(0.0)	1	(0.6)	1	(0.7)	4	(1.5)	1	(0.3)	1	(0.3)	-
Others													
Amantadine	33	(30.0)	41	(26.6)	42	(29.2)	53	(20.2)	69	(21.9)	78	(22.1)	0.029
Selegiline	12	(10.9)	20	(13.0)	21	(14.6)	33	(12.5)	40	(12.7)	37	(10.5)	0.528
Droxidopa	9	(8.2)	12	(7.8)	15	(10.4)	19	(7.2)	16	(5.1)	16	(4.5)	0.021
Entacapone	0	(0.0)	0	(0.0)	4	(2.8)	17	(6.5)	31	(9.8)	31	(8.8)	<0.001
Zonisamide	0	(0.0)	0	(0.0)	0	(0.0)	0	(0.0)	3	(1.0)	11	(3.1)	<0.001

a Levodopa alone and combination of levodopa with dopa-decarboxlyase inhibitor.

b Other anticholinergics include piroheptine, profenamine, and mazaticol.

c Cochran-Armitage trend test was used to calculate P-values (Statistical significance level was set at *P*<0.002 after Bonferroni correction).

The total proportion of prescribed anti-Parkinson drugs is not 100% due to the presence of plural drug users.

We further compared the selection of first-line drugs before and after April 2007, when the regulatory advisories were issued. This analysis (n = 414; 47 in pre-revision and 367 in post-prevision) revealed that the proportion of patients prescribed non-ergot dopamine agonists significantly increased (6.4% to 29.4%, *P*<0.001, Table 2), whereas the proportion of patients prescribed cabergoline or pergolide decreased, but not significantly (12.8% to 7.1%, *P* = 0.239). L-dopa was the most frequently prescribed first-line drug throughout the study period (42.6% to 42.2%). The proportion of patients prescribed anticholinergics as the first-line drug remained almost the same (27.7% to 27.0%).

**Table 2:** Proportions of First-line Drugs Prescribed Before and After Cabergoline and Pergolide Product Label Revisions for Valvulopathy Risk.

Anti-Parkinson drugs	Pre-revision (n = 47) (%)		Post-revision (n = 367) (%)		*P*-value
Age, mean [SD] (years) (median, IQR)^a^	57.5 [16.3] (56, 44–71)		55.4 [13.7] (56 44–64)		
Gender (men), N (%)	20	(42.6)	178	(48.5)	-
L-dopa	20	(42.6)	155	(42.2)	0.967
Ergot dopamine agonists	8	(17.0)	42	(11.4)	0.269
Cabergoline	5	(10.6)	17	(4.6)	0.090^c^
Pergolide	1	(2.1)	9	(2.5)	1.000^c^
Cabergoline or pergolide	6	(12.8)	26	(7.1)	0.239^c^
Bromocriptine	2	(4.3)	16	(4.4)	1.000^c^
Non-ergot dopamine agonists	3	(6.4)	108	(29.4)	<0.001
Pramipexole	3	(6.4)	84	(22.9)	0.009
Ropinirole	0	(0)	21	(5.1)	0.151^c^
Talipexole	0	(0)	5	(1.4)	1.000^c^
Anticholinergics	13	(27.7)	99	(27.0)	0.921
Trihexyphenidyl	8	(17.0)	69	(18.8)	0.768
Biperiden	5	(10.6)	28	(7.6)	0.404
Other anticholinergics^b^	0	(0)	2	(0.5)	-
Others	9	(19.1)	88	(24.0)	-
Selegiline	2	(4.3)	19	(5.2)	1.000^c^
Amantadine	5	(10.6)	58	(15.8)	0.378
Entacapone	0	(0)	13	(3.5)	0.378^c^
Droxidopa	2	(4.3)	7	(1.9)	0.272^c^
Zonisamide	0	(0)	0	(0)	-

“Pre-revision”: between July 2005 and March 2007. “Post-revision”: between April 2007 and December 2010.

a Age when a first-line drug was prescribed.

b Other anticholinergics include piroheptine, profenamine, and mazaticol.

Pearson's chi-square test was used to calculate P-values unless otherwise noted (*P*<0.002 after Bonferroni correction).

c Fisher's exact test.

**Figure 2:**
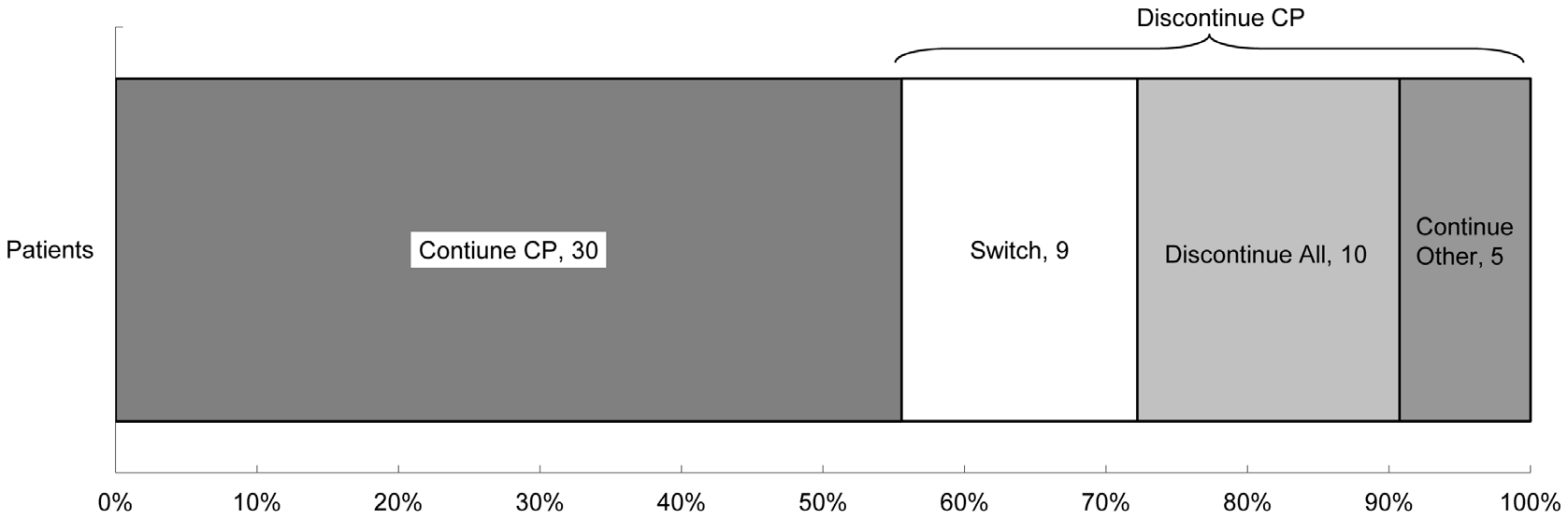
Prescription changes among cabergoline/pergolide user in one year before and after the 2007 measures. Continue CP: Continued cabergoline or pergolide until the end of the study period. Switch: Switched to other anti-Parkinson drugs. Discontinue All: Discontinued all anti-Parkinson drugs at once. Continue Other: Continued other anti-Parkinson drugs that had been co-prescribed. Nine patients switched from cabergoline or pergolide to other anti-Parkinson drugs (seven to pramipexole, one to ropinirole, and one to talipexole) during the period before April 2007 (three) and after April 2007 (six). Twenty-three patients were prescribed cabergoline or pergolide before April 2007 and continued after the 2007 revision: 18 were in the Continue CP group, four were in the Switch group, and one was in the Discontinue All group.

With respect to cabergoline/pergolide users one year before or after April 2007, 78 patients were prescribed cabergoline or pergolide between April 2006 and March 2008. Among them, 24 patients were excluded because they dropped out from the database before the end of the study period (two deaths were confirmed). The mean age of the dropout patients (seven men, 29.2%) was 69.4 years (standard deviation, 12.3), and the mean age of the selected 54 patients (28 men, 51.9%) was 57.4 years (standard deviation, 13.9). Thirty (55.6%) patients were continued on cabergoline or pergolide until the end of the study period (Figure 2). Of the remaining 24 (44.4%) patients who discontinued these drugs, nine (20.4%) switched to other anti-Parkinson drugs (pramipexole, seven; ropinirole, one; talipexole, one). During the same period, two of the 67 non-ergot agent users switched to ergot agents.

## Discussion

In this study, we examined trends of anti-Parkinson drugs prescribed to PD patients from 2005 to 2010 in Japan, and found that L-dopa was the most frequently used and was prescribed to more than half of the patients during the study period. After the product label revisions of ergot agents in 2007 to include a warning about the risk of valvulopathy, there was a decrease in the prescription of these drugs and an increase in the prescription of non-ergot agents.

### Prescribing trends for anti-Parkinson drugs

The proportion of patients prescribed L-dopa was approximately half of the PD patient population in this study. This suggests that L-dopa was the mainstay of PD treatment between 2005 and 2010, despite the introduction of new anti-Parkinson drugs. While the introduction of new non-ergot agents may have reduced the prescription of ergot agents, it had little influence on the prescription of L-dopa. Proportions of patients prescribed entacapone and zonisamide, two drugs commonly used together with L-dopa, were low, despite the slight increase observed after their release in 2007 and 2009. Moreover, prescription of these drugs had little influence on the proportion of L-dopa users.

In general, anticholinergics are not recommended for the treatment of PD, especially as first-line therapy, given their limited efficacy and neuropsychiatric side effects [7], [8], [29]. Yet, anticholinergics were prescribed to nearly 30% of new PD patients as first-line therapy, and were used in approximately 30% of all PD patients in this study. Compared to other countries [5], [30], [31],anticholinergics are generally preferred by Japanese clinicians, possibly due in part to Japanese clinical practice guidelines (2002 edition) which include anticholinergics as an option for early-stage PD patients [32]. Use of anticholinergics is expected to decrease in the future due to its exclusion as a recommended first-line drug in the 2011 edition of the clinical guidelines [7].

### Changes in the proportion of patients prescribed dopamine agonists after 2007

Between January 2005 and December 2010, the use of ergot dopamine agonists gradually decreased from 2005, but the use of non-ergot dopamine agonists soared since 2007. Ooba et al., using the same database that was used in the present study, focused on prescription patterns of cabergoline/pergolide and found no decrease between January 2005 and October 2008 [14]. The survey period of that study, compared with ours, may have been too short for the dissemination of safety information among clinicians, given that regulatory actions were taken in April 2007.

With respect to first-line drugs, we found that non-ergot agent use increased after April 2007. Given the many potential factors that may have influenced prescription behavior, we cannot determine the direct effects of the regulatory actions from this analysis. Nonetheless, the priority to prescribe non-ergot agents as the first-line drug might have been indirectly associated with the revised product labels of cabergoline and pergolide, which recommend that these drugs be administered to patients only when non-ergot agonists fail to show favorable effects [12]. Our findings imply that the revised product labels might be effective in modifying the prescribing behavior for new PD patients. About cabergoline/pergolide user analysis, more than half of cabergoline/pergolide users continued these drugs after the regulatory actions in 2007, but all switched patients were prescribed non-ergot agents. In contrast, only two patients prescribed non-ergot agents were switched to ergot agents during the same period. Other than the regulatory actions aimed at ergot agents, various factors may have contributed to the increased prescription of non-ergot agents, such as the sales promotion of non-ergot agents (beginning in 2004 for pramipexole and in 2006 for ropinirole). It is unlikely that clinical practice guidelines had any effect, given that they were published in 2002 and revised in 2011 [7], [32].

Drug utilization studies, such as the present one, have been widely used to monitor the effects of regulatory actions [33]–[35]. Some studies reported that information on drug safety did little to influence the prescription behaviors of clinicians [36]–[38], while others demonstrated the effectiveness of regulatory actions which included direct intervention for prescribers and attracted media attention [39]–[41]. Our results suggest that the regulatory actions directed at cabergoline and pergolide were possibly effective, although there was a delay of about 2 years before the effects became apparent.

### Limitations

This study has some limitations worth noting. First, the examined population in the present study was not representative of the whole population of Japan because the database only included employees of large companies and their family members; no employees of small companies, the self-employed, or retirees were included. Thus, generalization of the results requires prudent consideration. Second, the proportion of people aged 65 years and older was small, although PD occurs mainly in the elderly population [1], [2], [42]. Moreover, a different insurance system, which was implemented in 2008, applies to people aged 75 years and older [23]. Thus, the present findings reflected more middle-aged patients than elderly patients, particularly after 2008. To examine the possible age influence, we divided the patients by age 65 and compared the two groups (Table S1, S2). Our trend and first-line analyses showed that the proportion of L-dopa was higher, and that of anticholinergics lower, in the elderly group compared to the middle-aged group. These findings may be attributed to the 2002 practice guidelines, which recommend L-dopa but not anticholinergics for use in the elderly given the risk of neuropsychiatric adverse events [32]. Thus, the proportion of L-dopa might have been higher, and that of anticholinergics lower, had more elderly people been included in the present study. Furthermore, analyses showed moderate trends of decrease in ergot agents and increase in non-ergot agents in the elderly group. In the first-line analysis, proportion of non-ergot agents did not significantly increase in the elderly group. Consequently, findings on the use of dopamine agonists are not subject to serious bias, since there is no evidence of age-specific prescription patterns. Third, the reasons for dropout from the database could not be determined. Among patients prescribed cabergoline or pergolide in the year before or after April 2007, dropout patients were generally older than followed-up patients. Because elderly people are at a higher risk of valvulopathy than middle-aged people, the dropout group may have included more valvulopathy patients. Since the product labels of these two drugs were revised to include a contraindication against patients with a history of valvulopathy, clinicians might have considered switching ergot agents for elderly patients with valvulopathy to non-ergot agents. The dropout patients, if followed-up, might have included more elderly patients with a higher risk of valvulopathy, and thus the proportion of the switch group (from ergot to non-ergot agents) might have been higher. Therefore, the present finding that nine of 54 cabergoline/ pergolide user were switched to non-ergot agents may be an underestimation. Fourth, the data in the database has not been validated, except for the definition of death [43]. Therefore, it is possible that differences exist between database and actual clinical diagnoses. For example, some patients who were prescribed anticholinergics and classified as PD in the claim data may have had diseases other than PD. Indeed, anticholinergics are often prescribed to patients taking anti-psychotics to treat drug-induced parkinsonism [7], [44], [45]. Accordingly, we excluded patients who were prescribed both anti-Parkinson drugs and dopamine antagonists from the trend and first-line analyses, given the likelihood that they have drug-induced Parkinsonism. Finally, medical records before January 2005 were not available. Thus, based on incident user design [26], [27], patients who were diagnosed as PD or prescribed anti-Parkinson drugs within six months after the initial claim data were excluded from the first-line analysis, since it is unlikely that PD patients would interrupt their medication for more than six months. Therefore, there was a low probability that the present first-line analysis included those other than new PD patients.

In conclusion, L-dopa was the most frequently used drug to treat PD patients, being used by half of the PD patient population of this study, and anticholinergics were also frequently used in Japan between 2005 and 2010. Prescriptions of ergot agents decreased and those of non-ergot agents increased after the label revisions in 2007. Our findings highlight the need for clinicians and pharmacists to be conscious of drug safety information, and regulatory agencies and the pharmaceutical industry to continue monitoring the use and side effects of anti-Parkinson drugs.

## Supporting Information

Table S1 **Proportions of Parkinson's Disease Patients Prescribed Anti-Parkinson Drugs According to Two Age Groups.**
(DOC)

Table S2 **Proportions of Patients in Two Age Groups Who Were Prescribed First-line Drugs Before and After Cabergoline and Pergolide Product Label Revisions for Valvulopathy Risk.**
(DOC)
