# Correction: Prescribing Pattern of Anti-Parkinson Drugs in Japan: A Trend Analysis from 2005 to 2010

**DOI:** 10.1371/journal.pone.0106217

**Published:** 2014-08-19

**Authors:** The PLOS ONE Staff

There are formatting errors in Table S1. Please see the corrected Table S1 here.

## Supporting Information

Table S1 Proportions of Parkinson's Disease Patients Prescribed Anti-Parkinson Drugs According to Two Age Groups.
(DOC)
