# Supplementary material for: Prescribing Pattern of Anti-Parkinson Drugs in Japan: A Trend Analysis from 2005 to 2010
Source: PLoS One. 2014 Jun 6;9(6):e99021. doi: 10.1371/journal.pone.0099021 (PMC4048287; doi:10.1371/journal.pone.0099021)
Supplement: Table S1 — Proportions of Parkinson's Disease Patients Prescribed Anti-Parkinson Drugs According to Two Age Groups. (DOC) [file pone.0099021.s001.doc]

**Table S1. Proportions of Parkinson’s Disease Patients Prescribed Anti-Parkinson Drugs According to Two Age Groups**

| Age | Variable | | | 2005 | | 2006 | | 2007 | | 2008 | | 2009 | | 2010 | | *P*-value ^c^ |
| --- | --- | --- | --- | --- | --- | --- | --- | --- | --- | --- | --- | --- | --- | --- | --- | --- |
| < 65 | Number of patients | | | 69 | | 82 | | 75 | | 164 | | 245 | | 287 | | - |
|  | Age, median, IQR | | | 55, 46-59 | | 54, 44-59 | | 52, 44-60 | | 53, 43-60 | | 53, 44-60 | | 53, 44-59 | | - |
|  | Gender (male), N (%) | | | 43 | (62.3) | 53 | (64.6) | 51 | (68.0) | 108 | (65.9) | 139 | (56.7) | 161 | (56.1) | - |
|  | Parkinson’s disease duration (months) median, IQR | | | 30, 9-58 | | 31, 10-63 | | 39, 12-67 | | 28, 7-61 | | 21, 6-51 | | 21, 5-47 | | - |
|  | Category of drugs (%) | L-dopa ^a^ | | 37 | (53.6) | 38 | (46.3) | 33 | (44.0) | 69 | (42.1) | 106 | (43.3) | 126 | (43.9) | 0.246 |
|  |  | Ergot dopamine agonists | | 32 | (46.4) | 39 | (47.6) | 29 | (38.7) | 43 | (26.2) | 46 | (18.8) | 43 | (15.0) | < 0.001 |
|  |  | Non-ergot dopamine agonists | | 6 | (8.7) | 11 | (13.4) | 12 | (16.0) | 54 | (32.9) | 90 | (36.7) | 103 | (35.9) | < 0.001 |
|  |  | Anticholinergics | | 23 | (33.3) | 27 | (32.9) | 25 | (33.3) | 59 | (36.0) | 80 | (32.7) | 100 | (34.8) | 0.826 |
|  |  | Others ^b^ | | 28 | (40.6) | 29 | (35.4) | 33 | (44.0) | 52 | (31.7) | 89 | (36.3) | 94 | (32.8) | 0.195 |
| ≥ 65 | Number of patients | | | 41 | | 72 | | 69 | | 99 | | 70 | | 66 | | - |
|  | Age, median, IQR | | | 72, 69-83 | | 76, 71-82 | | 76, 72-84 | | 74, 71-81 | | 71, 67-73 | | 72, 68-73 | | - |
|  | Gender (male), N (%) | | | 12 | (29.3) | 18 | (25.0) | 17 | (24.6) | 12 | (29.3) | 18 | (25.0) | 17 | (24.6) | - |
|  | Parkinson’s disease duration (months) median, IQR | | | 22, 12-72 | | 29, 13-57 | | 37, 13-69 | | 33, 12-71 | | 42, 11-74 | | 33, 13-62 | | - |
|  | Category of drugs (%) | | L-dopa ^a^ | 27 | (65.9) | 47 | (65.3) | 43 | (62.3) | 75 | (75.8) | 52 | (74.3) | 54 | (81.8) | 0.009 |
|  |  |  | Ergot dopamine agonists | 12 | (29.3) | 13 | (18.1) | 9 | (13.0) | 15 | (15.2) | 11 | (15.7) | 4 | (6.1) | 0.006 |
|  |  |  | Non-ergot dopamine agonists | 4 | (9.8) | 8 | (11.1) | 7 | (10.1) | 26 | (26.3) | 29 | (41.4) | 22 | (33.3) | < 0.001 |
|  |  |  | Anticholinergics | 5 | (12.2) | 14 | (19.4) | 12 | (17.4) | 18 | (18.2) | 13 | (18.6) | 11 | (16.7) | 0.775 |
|  |  |  | Others ^b^ | 21 | (51.2) | 33 | (45.8) | 34 | (49.3) | 39 | (39.4) | 35 | (50.0) | 32 | (48.5) | 0.917 |

^a^Levodopa alone and combination of levodopa with dopa-decarboxlyase inhibitor.

^b^Others include amantadine, selegiline, droxidopa, entacapone, and zonisamide.

^c^Cochran-Armitage trend test was used to calculate P-values (statistical significance level was set at *P* < 0.002 after Bonferroni correction).

The total proportion of prescribed anti-Parkinson drugs is not 100% due to the presence of plural drug users.
