# Supplementary material for: Prescribing Pattern of Anti-Parkinson Drugs in Japan: A Trend Analysis from 2005 to 2010
Source: PLoS One. 2014 Jun 6;9(6):e99021. doi: 10.1371/journal.pone.0099021 (PMC4048287; doi:10.1371/journal.pone.0099021)
Supplement: Table S2 — Proportions of Patients in Two Age Groups Who Were Prescribed First-line Drugs Before and After Cabergoline and Pergolide Product Label Revisions for Valvulopathy Risk. (DOC) [file pone.0099021.s002.doc]

**Table S2. Proportions of Patients in Two Age Groups** Who Were Prescribed First-line Drugs Before and After Cabergoline and Pergolide Product Label Revisions for Valvulopathy Risk

| Age | Periods | N | Age, mean [SD] (years) (median, IQR) ^a^ | Gender (men),  N (%) | | L-dopa (%) | | Ergot dopamine  agonists (%) | | Non-ergot dopamine  agonists (%) | | Anticholinergics (%) | | Others ^b^  (%) | |
| --- | --- | --- | --- | --- | --- | --- | --- | --- | --- | --- | --- | --- | --- | --- | --- |
| < 65 | Pre-revision | 30 | 47.4 [9.8] (48, 40-55) | 16 | (53.3) | 9 | (30.0) | 7 | (23.3) | 2 | (6.7) | 11 | (36.7) | 5 | (16.7) |
|  | Post-revision | 277 | 49.6 [9.9] (51, 41-59) | 125 | (54.9) | 99 | (35.7) | 34 | (12.3) | 85 | (30.7) | 85 | (30.7) | 60 | (21.7) |
|  | *P*-value |  |  |  | | 0.532 | | 0.096 ^c^ | | 0.006 | | 0.502 | | 0.525 | |
| ≥ 65 | Pre-revision | 17 | 75.3 [7.4] (74, 70-80) | 4 | (23.5) | 11 | (64.7) | 1 | (5.9) | 1 | (5.9) | 2 | (11.8) | 4 | (23.5) |
|  | Post-revision | 90 | 73.3 [6.2] (72, 70-74) | 26 | (28.9) | 56 | (62.2) | 8 | (8.9) | 23 | (25.6) | 14 | (15.6) | 28 | (31.1) |
|  | *P*-value |  |  |  | | 0.846 | | 1.000 ^c^ | | 0.112 ^c^ | | 1.000 ^c^ | | 0.531 | |

“Pre-revision”: between July 2005 and March 2007. “Post-revision”: between April 2007 and December 2010.

^a^ Age when a first-line drug was prescribed.

^b^ Others include amantadine, selegiline, droxidopa, entacapone, and zonisamide.

Pearson’s chi-square test was used to calculate P-values unless otherwise noted (*P* < 0.002 after Bonferroni correction).

^c^ Fisher’s exact test.
